# Solvent-Driven Nanostructural Tuning of Lignin/Poly(*N*,*N*-dimethylacrylamide) Hydrogels

**DOI:** 10.3390/gels12040277

**Published:** 2026-03-26

**Authors:** Xiaoqing Jiang, Xiangyu You, Xinhong Li, Ruiyun Tian, Xuelian Wang, Pedram Fatehi, Kang Kang, Xulong Zhu, Huijie Zhang

**Affiliations:** 1 College of Bioresources Chemical and Materials Engineering, Shaanxi University of Science & Technology, Xi’an 710021, China; 2 School of Food and Liquor Engineering, Sichuan University of Science & Engineering, Yibin 644000, China; 3 Green Processes Research Centre and Department of Chemical Engineering, Lakehead University, Thunder Bay, ON P7B 5E1, Canada; 4 The Key Laboratory of Biomedical Information Engineering of Ministry of Education, School of Life Science and Technology, Xi’an Jiaotong University, Xi’an 710049, China; zhuxulong@stu.xjtu.edu.cn

**Keywords:** lignin, hydrogel, lignin aggregation, nanostructural transformation, energy dissipation

## Abstract

Non-covalent molecular self-assembly serves as a distinctive strategy for enhancing the mechanical performance of lignin-based composite hydrogels. Nevertheless, the self-assembly process can be significantly influenced, leading to alterations in the nanostructure of the hydrogel, because of the diverse conformational reorganizations of lignin in different solvents. In this research, a solvent exchange process was employed to generate a phase-separated structure comprising hydrophobic lignin domains and hydrophilic poly(*N*,*N*-dimethylacrylamide) (PDMA) domains through the aggregation of lignin, thereby forming tough lignin/PDMA hydrogels. By adjusting the solvent composition, the hydrogels exhibit distinct nanostructural transformations that are precisely correlated with the changes in Hansen Solubility Parameters (HSPs) of the solvent mixtures. Balanced HSPs facilitates the formation of small-scale lignin domains with high-domain density, which act as crosslinking points for the establishment of a reinforced network. Remarkably, lignin/PDMA hydrogels prepared at a boundary solvation condition unexpectedly induced the formation of large and highly condensed lignin domains, which displayed a radius of gyration (*R*g) of 7.7 nm and an inter-domain distance (*d*-spacing) of 98.1 nm within the hydrogel network. These unique nanostructural features further contribute to its superior mechanical performance, including excellent tensile strength of 3.2 MPa, Young’s modulus of 5.7 MPa, and fracture energy of 41.2 kJ m^−2^, which outperforms most reported lignin hydrogels. Additionally, it offers a strong adhesion and rapid drying approach, rendering the hydrogel more suitable for applications as hydrogel coatings.

## 1. Introduction

Most hydrogels, particularly those based on biopolymers, frequently exhibit suboptimal mechanical properties, and this constraint significantly impedes their practical utilization [1,2,3]. To address these mechanical defects, various cross-linking combinations (e.g., chemical cross-linking [4,5], thermal condensation [6,7], ionic gelation [8], electrostatic interaction [9,10]) as well as network design strategies like slide-ring [11] and double-network [12] architectures have been explored to improve toughness. Among these, non-covalent molecular self-assembly represents a distinctive approach, especially for biomacromolecules [13,14,15]. It employs weak non-covalent bonds, including hydrogen bonds, van der Waals forces, hydrophobic interactions, etc., to direct macromolecules into well-defined scaffolds with improved structure and functionality [3]. A notable example is the hierarchical self-assembly of cellulose. Individual cellulose chains initially self-assemble to form nanocrystalline regions, and these nanocrystals subsequently act as building units to construct larger and more complex fibrous structures present in plants. Such a structure endows cellulose fibers with remarkable tensile strength and stiffness, which are far more superior than those of an individual cellulose chain [16,17].

Lignin, the second most abundant renewable bioresource in nature, has predominantly been employed for energy recovery via combustion. As a promising valorization of lignin, lignin-based hydrogels have garnered substantial attention [18,19,20]. This approach not only facilitates the value-added utilization of lignin but also endows the hydrogels with distinctive functionalities, including antioxidant activity [21,22], UV resistance [23,24], and adhesive properties [25,26]. Despite these advancements, conventional lignin hydrogels also exhibit poor mechanical performance, particularly low toughness (fracture energy typically below 10 kJ·m^−2^), owing to their brittle and complex structure, which makes it difficult to meet the practical demands of load-bearing or high-strength applications [1,2]. Inspired by the hierarchical self-assembly of cellulose, our previous research establishes the feasibility of employing non-covalent self-assembly strategies in lignin-based hydrogels to address their mechanical limitations [27]. By utilizing a specific dry–swelling solvent exchange method, lignin molecules and poly (*N*,*N*-dimethylacrylamide) (PDMA) chains self-assembled to form a network. This network comprises hydrophobic lignin-rich domains and hydrophilic PDMA domains, which endows the hydrogel with efficient energy dissipation under tensile deformation. As a result, a lignin-based hydrogel with an excellent fracture energy of up to 16,000 J m^−2^ was achieved.

Nevertheless, as a complex biopolymer, lignin has a considerably more intricate structure than that of synthetic polymers and polysaccharides. Notably, lignin exhibits distinct conformations and sizes in different solvents. Harton et al. reported that the radius of gyration (*R*g) of lignin molecules can be influenced by extraction and modification methods, with values ranging from 2.2 nm to 2.8 nm [28]. Analogously, Yue et al. found that different solvents can induce diverse conformational reorganizations of lignin molecules [29]. According to the Hansen Solubility Parameters (HSP) theory, lignin solubility depends on the HSP distance (*Ra*) between lignin and a given solvent within the Hansen space [30]. Therefore, alterations in the affinity within a self-assembled hydrogel system, which are caused by these solubility disparities, may affect the structure and even the mechanical performance of the resultant hydrogels. However, the specific influence of solvents on the self-assembled structures and the mechanical properties of resultant hydrogels is rarely reported.

**Scheme 1:**
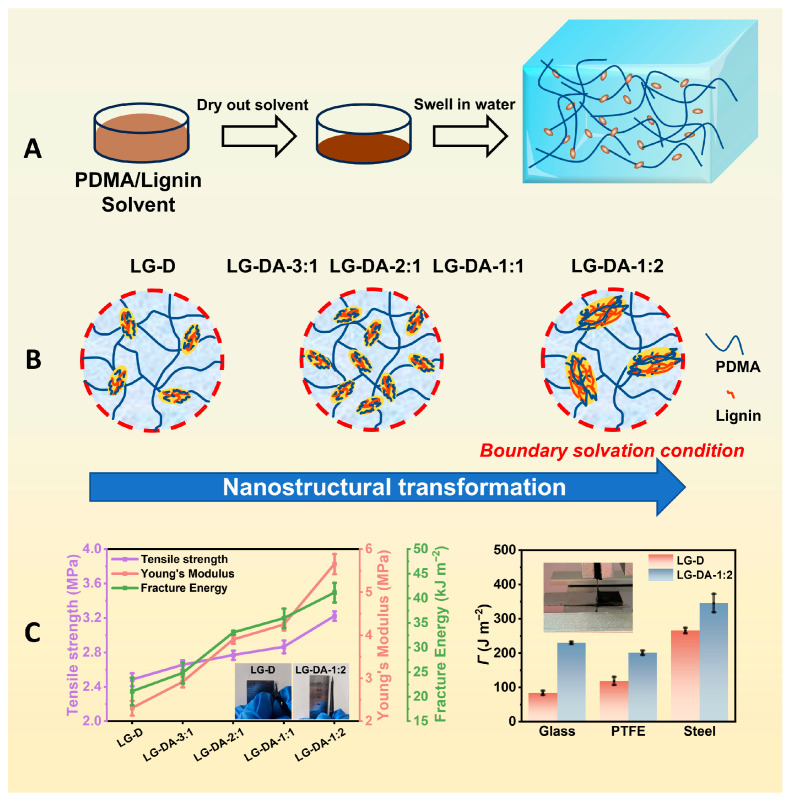
Schematic overview of lignin/PDMA hydrogel design and performance: (**A**) preparation process, (**B**) nanostructural transformations, and (**C**) demonstration of tensile and adhesive properties.

In this study, a binary solvent system of *N*,*N*-dimethylformamide (DMF)/acetone was used to prepare solutions with different solubilities of lignin. Tough lignin/PDMA composite hydrogels were fabricated through the drying–swelling solvent exchange method (Scheme 1). Specifically, lignin and PDMA were initially dissolved in the solvents, followed by a drying step for self-assembly, and ultimately swollen in water. It was discovered that the alterations in the solubility of lignin in the binary solvents influenced both the size and the distance of the aggregated lignin domains. These structural changes, in turn, had an impact on the mechanical properties of the lignin/PDMA hydrogels. Moreover, the use of acetone with high volatility was expected to accelerate the hydrogel formation process, rendering the hydrogel more suitable for applications as hydrogel coatings.

## 2. Results and Discussion

### 2.1. Solubility of Lignin in Solvents

**Figure 1:**
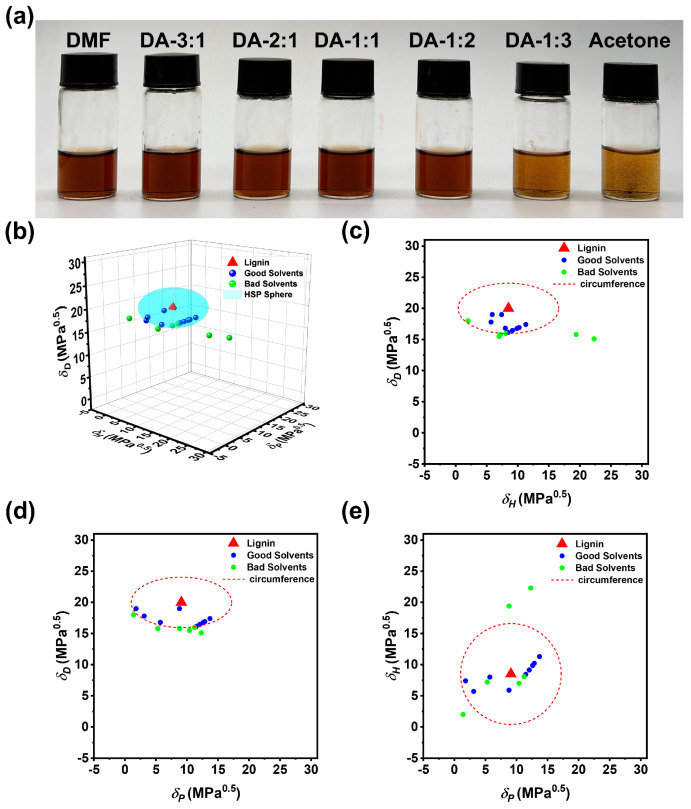
(**a**) Dissolution behavior of lignin in various solvents; (**b**–**e**) 3D and 2D representations of the HSP sphere for lignin.

As shown in Figure 1a, the solubility of lignin in different solvents is visually demonstrated, and detailed HSP values are summarized in Table S1. To estimate the HSP of lignin, the principle of “like dissolves like” was employed. Based on these experimental findings and using a desirability function [31], the HSP of our lignin was empirically approximated to be *δ_D_* = 20.0 MPa^0.5^, *δ_P_* = 9.1 MPa^0.5^, and *δ_H_* = 8.5 MPa^0.5^. A radius of an interaction sphere in Hansen space (*R*_0_) of 8.1 MPa^0.5^ was established as a threshold to distinguish between good and poor solvents for lignin (Figure 1b–e), which is consistent with the literature-derived *R*_0_ typically ranging from 6.5 to 10.5 MPa^0.5^ [31,32,33].

DMF is a favorable solvent for lignin, exhibiting *δ_D_*, *δ_P_*, and *δ_H_* values of 17.4, 13.7, and 11.3 MPa^0.5^, respectively. The low *Ra*/*R*_0_ value of 0.92 (less than 1) also indicates its feasibility for lignin dissolution [30]. However, acetone dissolves only a portion of lignin (*Ra*/*R*_0_ = 1.14). The large *δ_D_* difference between acetone (15.5 MPa^0.5^) and lignin (20.0 MPa^0.5^) indicates insufficient dispersion matching to effectively overcome non-polar interactions within the lignin molecules. Upon adding acetone to DMF, the HSP of the mixed solvents gradually shifted towards those of acetone (Table S1). When the DMF/acetone ratio was no lower than 1:2, the HSP of the mixed solvents fell within the solubility sphere of lignin (*Ra* = 7.42~7.72 MPa^0.5^ and *Ra*/*R*_0_ = 0.92~0.95).

It is worthy to note that the calculated values of *Ra*/*R*_0_ are the same when DMF, DA-3:1 and DA-2:1 were used as a solvent, but different *δ_D_*, *δ_P_*, and *δ_H_* of these solvents reflected their different dominant interactions to lignin [34]. Specifically, *δ_D_* characterizes the affinity of the solvent to the aromatic ring in lignin; *δ_P_* corresponds to the dipole–dipole interactions with -OCH_3_ and -C=O of lignin; and *δ_H_* represents the hydrogen bonding with -OH groups of lignin molecules. Thus, compared within these three solvents, the smaller *δ_D_* difference between DMF and lignin (Δ*δ_D_* = 2.6 MPa^0.5^) suggests a π-π stacking-dominated solvation while DA-3:1 and DA-2:1 prefer a polar and hydrogen bond-dominated solvation due to the smaller lignin–solvent difference in *δ_P_* and *δ_H_*, which might further result in different aggregation pathways and aggregate size of lignin during the solvent evaporation process in hydrogel preparation.

As a boundary condition at DA-1:2 (*Ra* = 8.10 MPa^0.5^ and *Ra*/*R*_0_ = 1.00), the solvation ability of the binary solvents declined, and the lignin molecules exhibited a slight tendency to aggregate. Considering that PDMA can be fully dissolved in water, DMF and acetone, whereas lignin can only be readily dissolved in DMF (Figure S1 and Figure 1a), the DMF/acetone system could lead to more pronounced size variations in lignin, which may further influence the energy-dissipative structures during hydrogel formation.

### 2.2. Structural Analysis of Lignin/PDMA Hydrogels

**Figure 2:**
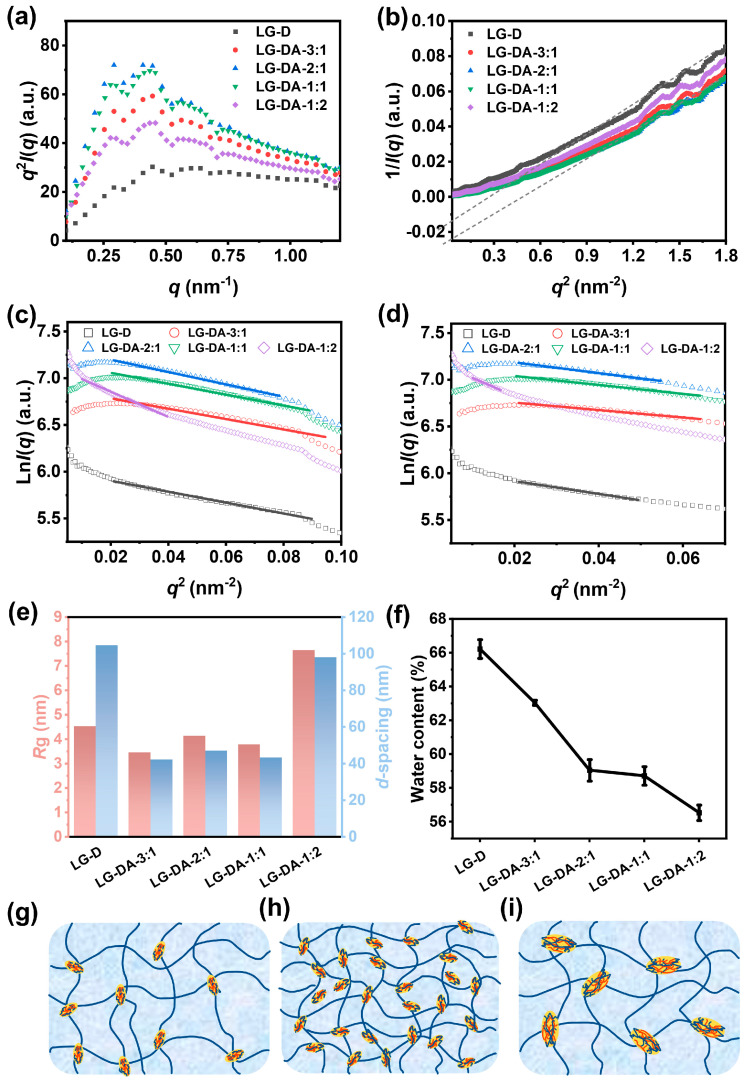
(**a**) Kratky plots; (**b**) Zimm plots; (**c**,**d**) Guinier plots (hollow symbols) and their corresponding fits (solid lines) within different *q* ranges: (**c**) *q*_min_*R*g < 0.65 and *q*_max_*R*g < 1.3; (**d**) *q*_min_*R*g < 0.65 and *q*_max_*R*g < 1.0; (**e**) comparison of *R*g and *d*-spacing values; (**f**) water content of lignin/PDMA hydrogels; structural illustrations of (**g**) LG-D, (**h**) LG-DA-3:1, LG-DA-2:1, LG-DA-1:1, and (**i**) LG-DA-1:2 (dark blue line, PDMA; brown line, lignin; orange area, lignin-rich region; light blue background, water).

To elucidate the impacts of solvent changes on the structural transformation of lignin/PDMA hydrogels, the structural characteristics of LG-DA-x:y were investigated using Small-angle X-ray Scattering (SAXS). The 1D *I*(*q*) vs. *q* profile derived from the scattering patterns exhibited shoulders in the low-*q* region (Figure S2a), suggesting non-homogeneous structures across all lignin/PDMA hydrogels. Subsequently, the SAXS patterns were transformed to obtain Kratky and Zimm plots. All Kratky plots reached a maximum and then decreased towards the horizontal asymptote (Figure 2a), indicating the presence of branching networks in all lignin/PDMA hydrogels [28,35]. This non-homogeneity was further verified by the deviation from linear behavior in the low-*q* region of the Zimm plots, where the negative intercepts signified the occurrence of phase separation in these hydrogels (Figure 2b) [35,36]. These characteristics are consistent with our established self-assembly mechanism in previous research using the same dissolve–dry–swell solvent exchange method: the nanostructure is formed through the hydrophobic self-assembly of lignins and the amphiphilic PDMA chains. During drying, lignin aggregates into dense lignin-rich domains, which subsequently act as physical cross-linking points when the PDMA-rich regions expand in the swelling process [27].

To quantitatively analyze the structural transformations in response to variations in the ratio of binary solvents, *R*g and *d*-spacing, representing the size of lignin domains and the distance between domains, respectively [27,37], were calculated based on Guinier plots and broad peak models. In the *q* range of Guinier plots where *q*_min_*R*g < 0.65 and *q*_max_*R*g < 1.3 (Figure 2c), the curves of LG-D and LG-DA-1:2 exhibited a characteristic upward trend at low *q*, suggesting an aggregation tendency of lignin molecules [38]. In contrast, the profiles of the remaining samples showed downturn, indicating repulsive interparticle interactions among lignins [38]. When the *q* range of Guinier plots was narrowed to *q*_min_*R*g < 0.65 and *q*_max_*R*g < 1.0 (Figure 2d), Ln(*I*(*q*)) showed linear dependence on *q*^2^ (*R*^2^ > 0.97, Table S2). This indicates an extended shape of lignin domains, and the *R*g values ranged from 3.5 nm to 7.7 nm (Figure 2e).

Additionally, the *I*(*q*) vs. *q* profile was effectively fitted using the broad peak model to determine the *d*-spacing between scattering objects (Figure S2b–f and Table S3) [39]. As depicted in Figure 2e, with increasing acetone content, the *d*-spacing initially decreased from 104.7 nm in LG-D to approximately 45.0 nm (LG-DA-3:1 to LG-DA-1:1), and then suddenly increased to 98.1 nm in LG-DA-1:2. These findings can be ascribed to the intricate interplay resulting from diverse HSP, which regulates the aggregation morphology of lignin domains.

For the LG-D sample with a water content of 66.2% (Figure 2f and Table S4), the closed *δ_D_* value between DMF and lignin was effective in disrupting some of the π-π stacking of lignin. However, the large difference in corresponding values of *δ_P_* and *δ_H_* were insufficient to overcome strong dipole–dipole interactions and hydrogen bonds within lignin molecules. This imbalanced interaction led to the formation of numerous small-scale lignin domains of approximately 4.5 nm, which tended to aggregate and were separated by a large distance of 104.7 nm (Figure 2g).

Conversely, the addition of acetone to the solvent for the LG-DA-3:1 to LG-DA-1:1 samples strategically adjusted the HSP values. Both *δ_P_* and *δ_H_* of the mixed solvents were aligned with the values of lignin, which promoted more efficient disruption of hydrogen bonds among lignins. In this case, more dipole end and hydroxyl groups were exposed in solvents, which hinders lignin aggregation due to the predominant repulsive interactions among lignin molecules. As a result, smaller lignin domains of about 4.0 nm and a significantly reduced domain spacing around 45.0 nm were formed (Figure 2h). Considering that lignin domains act as cross-linking points in hydrogels, the small-sized domains and high cross-linking density in the LG-DA samples gave a decreased water content ranging from 63.0% to 58.7% (Figure 2f and Table S4). Moreover, they might further result in higher mechanical performance compared to the LG-D sample.

When a high ratio of acetone was present, the lowest water content of 56.5% was achieved at LG-DA-1:2. Meanwhile, the overall solvent quality deteriorated significantly. The HSP of the binary solvents became poorly matched with those of lignin, leading to more severe aggregation. This significant desolvation drove lignin molecules to aggregate and self-assemble highly compacted domains with the largest *R*g value of 7.7 nm and a moderate *d*-spacing of 98.1 nm (Figure 2i). These structural features also endow a higher energy dissipation capability compared to LG-D.

### 2.3. Mechanical Performance and Coating Applications of Lignin/PDMA Hydrogels

**Figure 3:**
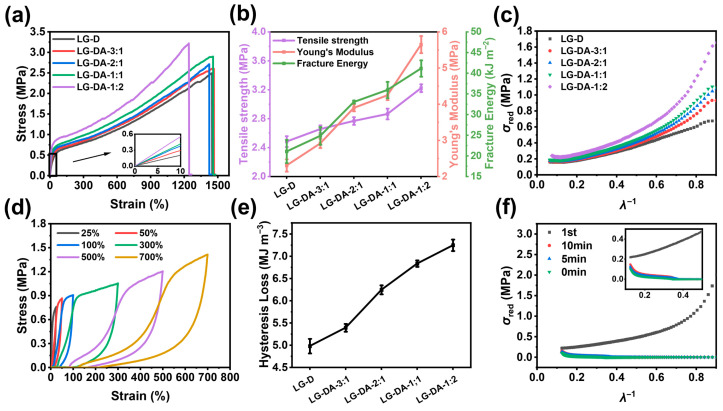
(**a**,**b**) Tensile curves and properties diagrams of lignin/PDMA hydrogels. Inset: magnified view of the low-strain region; (**c**) Mooney–Rivlin model fitting for lignin/PDMA hydrogels; (**d**) cyclic tensile curves of LG-DA-1:2 at different strains with no waiting time; (**e**) hysteresis loss of lignin/PDMA hydrogels at a strain of 700%; (**f**) Mooney–Rivlin model fitting for cyclic stretching of LG-DA-1:2. Inset: magnified view of the strain-hardening region.

The impact of different solvents on the mechanical properties of lignin/PDMA hydrogels was evaluated via uniaxial tensile tests and tear tests (Figure 3a,b and Table S5). The original LG-D hydrogel exhibited tough hydrogel characteristics, including a tensile strength of 2.5 MPa, a Young’s modulus of 2.3 MPa, and a tearing fracture energy of 21.1 kJ m^−2^. As discussed in our previous study, energy can be effectively dissipated by fractures of lignin domains by pulling out of the PDMA chains from the lignin domains and the complete fracture of these domains and even the hydrogel [27]. With increasing acetone content, the mechanical properties of LG-DA-3:1 to LG-DA-1:1 gradually increased. This was attributed to the higher cross-linking density resulting from the reduced lignin domain size (*R*g) and the shorter distance between lignin domains (*d*-spacing).

Optimal mechanical performance was achieved at LG-DA-1:2, with a tensile strength of 3.2 MPa, a Young’s modulus of 5.7 MPa, and a tearing fracture energy of 41.2 kJ m^−2^, which were 128.0%, 247.8%, and 195.3% of those of the original LG-D, respectively. The Mooney–Rivlin model was employed to further explore the tensile behaviors of the lignin/PDMA hydrogels. In the equation expression of *σ*_red_ = *σ*/(*λ* − *λ*^−2^) = 2*C*_1_ + 2*C*_2_/*λ*, *σ*_red_ is the reduced stress, *λ* is the stretch ratio, and *C*_1_ and *C*_2_ are the material constants [40,41]. Specifically, *C*_2_ is associated with strain hardening (*C*_2_ < 0) and/or softening (*C*_2_ > 0) beyond the Gaussian elasticity region, while *C*_2_ = 0 indicates that the material is in a purely elastic stretching region. As the plotted *σ*_red_ versus *λ*^−1^ curves shown in Figure 3c, the slopes, in terms of *C*_2_, were all greater than 0, indicating continuous strain softening of the lignin/PDMA hydrogels caused by the fracture of the aggregated lignin domains throughout the entire tensile process. The strain softening became more noticeable with an increase in the acetone amount due to the increased number density of the lignin domains. The maximum *C*_2_ value was observed in the LG-DA-1:2 sample, which contained the largest lignin aggregates. This indicates the most pronounced strain softening of the hydrogel during the deformation process, which is attributed to the fracture of the large lignin aggregates.

Cyclic tensile tests were further conducted to investigate the fracture process of the lignin aggregates. As shown in Figure 3d, significant hysteresis was observed across a wide range of strains from 25% to 700%, indicating continuous energy dissipation caused by the fracture of the lignin aggregates. Meanwhile, under the same maximum tensile strain of 700%, the area enclosed by the cyclic stress–strain curves reached its highest value of 7.2 MJ m^−3^ at LG-DA-1:2 (Figure S3 and Figure 3e). This finding further indicates that LG-DA-1:2 exhibited the highest energy dissipation capacity during deformation. These results are consistent with the analysis of the tensile stress–strain curve using the Mooney–Rivlin model. Furthermore, the stretched hydrogel exhibited irreversible mechanical performance from small to large tensile strains, without significant recovery observed even after resting for 10 min (Figure S4). This phenomenon is attributed to the hydrophobic nature of lignin, which is insoluble in water (Figure S5). Once the lignin-rich domains fracture during the initial loading, the surrounding water prevents their structural recovery, which is similar to the sacrificial bond mechanism in double-network hydrogels [12].

We conducted a more in-depth analysis of the second-round loading curves corresponding to different resting times using the Mooney–Rivlin model (Figure 3f). It was found that after the first loading, the *C*_2_ values of the tensile stress–strain curve were all approximately 0, indicating the Gaussian elasticity of LG-DA-1:2. Based on the analyses above, it is apparent that lignin aggregates serve as primary energy-dissipating structures in the hydrogel. These aggregates successively fractured throughout the entire tensile process, and the size and density of the lignin aggregates exerted an influence on the mechanical properties of the hydrogel. By optimizing the size and structure of the lignin aggregates via solvent adjustment, lignin hydrogels with outstanding mechanical performances can be obtained.

**Figure 4:**
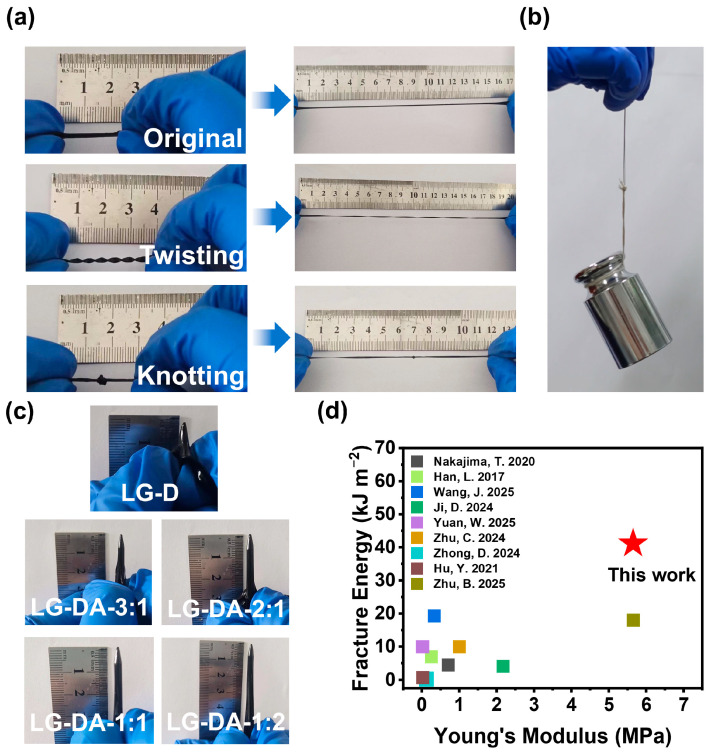
Visual demonstrations of (**a**) direct, twist, and knot stretching of LG-DA-1:2; (**b**) LG-DA-1:2 lifting a 500 g weight and (**c**) puncture resistance of lignin/PDMA hydrogels. (**d**) Fracture energy and Young’s modulus comparison of various hydrogels [1,2,42,43,44,45,46,47,48].

A series of practical experiments were conducted to explicitly demonstrate the outstanding performance of LG-DA-1:2. As depicted in Figure 4a,b, LG-DA-1:2 can endure various tensile deformations, including torsion, kinking, and lifting weights up to 500 g. In addition, when puncture resistance tests were performed, the LG-D exhibited the smallest puncture strain by nails approximately 1 cm in length (Figure 4c). As acetone content increased, the toughness of LG-DA samples significantly enhanced, and the largest deformation of 5.5 cm was achieved at LG-DA-1:2. Therefore, these results indicate that LG-DA-1:2 is a tough hydrogel featuring an effective energy-dissipative structure, which is attributed to the large lignin domain size with proper distance among the lignin domains. In comparison with conventional single-network lignin hydrogels and even several reinforced tough composite hydrogels, LG-DA-1:2 demonstrated performance at least twice as high as that of hydrogels reported in previous studies (Figure 4d) [1,2,42,43,44,45,46,47,48].

**Figure 5:**
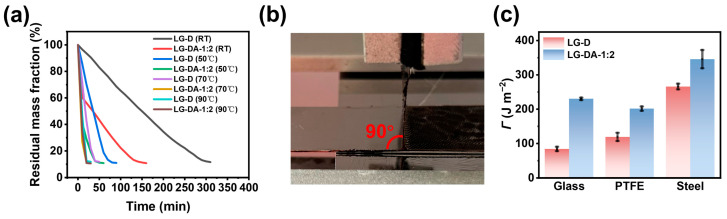
(**a**) Drying rate comparison of LG-D and LG-DA-1:2. (**b**) Photos of the 90° peeling test using a steel plate substrate. (**c**) Peeling energy of lignin/PDMA hydrogel coatings on different substrates.

An interesting application of LG-DA-1:2 lies in its utilization as a coating material. A total of 2 mL of a lignin/PDMA precursor solution was first added dropwise onto a steel plate and transferred to a hot stage to conduct drying rate measurement. As depicted in Figure 5a, LG-D maintained an approximately constant evaporation rate of about 0.2 g h^−1^ at room temperature and became completely dry after 5 h. Owing to the high vapor pressure of acetone, the drying process was accelerated to complete within 150 min. When the evaporation temperature gradually rose, the drying time was reduced to 20 min, which further reduced the cost associated with the lignin/PDMA hydrogel-based coating material in practical applications.

Moreover, the coating’s peel energy was tested through a 90° peel experiment (Figure 5b). LG-D exhibited peeling energies of 84.2 J m^−2^, 119.2 J m^−2^, and 266.0 J m^−2^ on glass, polytetrafluoroethylene (PTFE), and steel plate substrates, respectively (Figure 5c and Table S6). Notably, LG-DA-1:2 demonstrated higher values compared to those of LG-D and achieved the highest performance of 345.8 J m^−2^ on the steel substrate. This is because LG-DA-1:2 exhibits superior toughness around the adhesion interface, enabling it to undergo extensive deformation and dissipate a significant amount of energy. This characteristic substantially contributes to the interfacial toughness and cooperatively enhances the overall peeling energy [49]. Therefore, these findings highlight the considerable potential of LG-DA-1:2 for coating applications.

## 3. Conclusions

In summary, super tough lignin/PDMA hydrogels with tunable nanostructures were fabricated using binary solvents. Owing to the distinct HSPs of DMF/acetone solvents at varying ratios, lignin molecules exhibited different interaction tendencies, which further gave rise to different lignin domain sizes and domain distances in the prepared lignin/PDMA hydrogels. Specifically, for samples ranging from LG-DA-3:1 to LG-DA-1:1 with optimized HSP, a densely crosslinked network with small *R*g of 4.0 nm and a low *d*-spacing of 45.0 nm was formed, characterized by repulsive interactions among lignin molecules. In contrast, LG-DA-1:2, as a boundary condition, resulted in the formation of larger-scale lignin domains with a size of 7.7 nm and a moderate *d*-spacing of 98.1 nm due to the aggregative interactions among lignin molecules which dissipated energy more effectively. As a result, LG-DA-1:2 demonstrated the most superior mechanical performance, including an excellent tensile strength of 3.2 MPa, a Young’s modulus of 5.7 MPa, and a fracture energy of 41.2 kJ m^−2^, outperforming most reported lignin hydrogels. Additionally, LG-DA-1:2 also exhibited strong adhesion and rapid drying characteristics, suggesting its significant application potential in hydrogel coatings.

## 4. Materials and Methods

Materials: Acetic acid lignin (Mw of 5.9 kDa, PDI of 1.6, obtained using the calibration curve of polystyrene) extracted from bamboo was kindly provided by Guangzhou Yinnovator Biotech Co., Ltd., Guangzhou, China. *N*,*N*-Dimethylacrylamide (DMA), α-ketoglutaric acid, DMF, and acetone were purchased from Sinopharm Chemical Reagent Co., Ltd., Shanghai, China. All reagents were used as received.

HSP Determination of lignin: A precise quantity of 2.5 mg of lignin was weighed and combined with 5 mL of diverse solvents in glass vials. Following 6 h of shaking to facilitate maximum dissolution, the solubility was assessed via visual inspection. Specifically, a soluble sample manifested no solid residues, no turbidity, and presented a clear dark-colored solution. The HSPs of lignin were inferred from the outcomes of the solubility investigation utilizing the Microsoft Excel tool (Microsoft Excel 2021) developed by Diaz de los Rios and Hernandez Ramos [31]. The HSP distance, *Ra*, between lignin and a specific solvent was calculated by the following equation:

(1) $${\left(Ra\right)}^{2}=4{\left(\delta_{D2}-\delta_{D1}\right)}^{2}+{\left(\delta_{P2}-\delta_{P1}\right)}^{2}+{\left(\delta_{H2}-\delta_{H1}\right)}^{2},$$

where *δ_D_*, *δ_P_* and *δ_H_* represent the dispersion, polar and hydrogen bonding solubility parameters, respectively. Subscripts 1 and 2 refer to the solvent and lignin, respectively.

Preparation of lignin/PDMA hydrogels: Lignin/PDMA hydrogel was synthesized via a solvent exchange process in accordance with our previously reported method [27]. Specifically, 10 mL of a 1 wt% DMA aqueous solution containing α-ketoglutaric acid (0.2 mol% of DMA) as a photoinitiator was polymerized under UV irradiation for 6 h, followed by drying to yield a solid PDMA precursor. Subsequently, the PDMA precursor and lignin with a mass ratio of 8:11 were dissolved in various organic solvents with 1 h of stirring to obtain homogeneous lignin/PDMA precursor solutions. Finally, the precursor solution was thoroughly dried at 60 °C to eliminate the organic solvents and then swollen in water to acquire the final hydrogel products. To distinguish the organic solvent sources of these hydrogels, they were designated as follows: LG-D, indicating hydrogels prepared using DMF; LG-DA-x:y represents the hydrogel prepared with binary solvents system of DMF and acetone, where x:y denotes the volume ratio of DMF to acetone.

Water Content Determination: To measure the water content, the hydrogels were weighed in their fully swollen and dried conditions. The water content (*M*) was computed by means of the following formula:

(2) $$M=\left(W_{0}-W_{1}\right)/W_{0}\times 100\%,$$

where *W*_0_ and *W*_1_ are the weights of the swollen and dried samples, respectively.

SAXS Analysis: SAXS measurements were performed on swollen lignin hydrogels using BL19U2 beamlines at the Shanghai Synchrotron Radiation Facility (SSRF) (Shanghai, China). The X-ray energy was set at 12 keV, and the distance from sample to detector was 2700 mm. The two-dimensional SAXS patterns were captured using a Pilatus 1m detector with a pixel size of 100 μm and a pixel array of 981 × 1043.

For SAXS data analysis, beam stop masking and background subtraction were conducted to obtain the one-dimensional (1D) SAXS profiles of hydrogel samples. The scattering vector (*q*) dependence of the scattering intensity (*I*(*q*)) was radially averaged and the results were presented using various plots: Kratky plot (*q*^2^*I*(*q*) vs. *q*), Zimm plot (1/*I* vs. *q*^2^), and Guinier plot (Ln(*I*(*q*)) vs. *q*^2^). In Guinier analysis, the linear fitting line was performed within *q*_min_*R*g < 0.65 and *q*_max_*R*g < 1.0 to ensure high linearity (*R*^2^ > 0.97), and *R*g was obtained from the slope *k* (*k* = −*R*g^2^/3). The range up to *q*_min_*R*g < 0.65 and *q*_max_*R*g < 1.3 was simultaneously monitored to evaluate potential inter-domain associations. Then, the 1D SAXS profiles (*I*(*q*) vs. *q*) were fitted with the broad peak model (*I*(*q*) = *C*/(1 + (|*q* − *q*_0_|*ξ*)*^m^*) + *B*) that characterizes a multiphase system. The inter-domain distance, *d-*spacing, was then calculated using *d* = 2π/*q*_0_, where *q*_0_ is the peak position corresponding to the highest scattering intensity.

Tensile Test: The mechanical properties of the hydrogel samples were assessed using a universal tensile testing machine (HZ-1007C, Dongguan Lixian Instruments, Dongguan, China) equipped with a 50 N load sensor at 22 °C. The hydrogel samples were cut into dumbbell-shaped specimens in line with the dimensions specified in the standard JIS K 6251 [50] (12 mm gauge length × 2 mm width × ≈0.7 mm thickness) for uniaxial tensile tests. The initial distance (*L*_0_) between the two fixtures of the testing machine was set at 12 mm, and tensile deformation was applied at a speed of 100 mm min^−1^.

Tear Test: Tearing tests were performed on rectangular hydrogel specimens (35 mm × 15 mm, with a 10 mm notch at the midpoint of the short side) using the same tensile testing machine. The specimen was clamped and stretched at a constant rate of 100 mm min^−1^, and the force (*F*) exerted for tearing was recorded. The tearing fracture energy (*T*) was calculated by the formula:

(3) T = 2F/W,

where *W* represents the thickness of the specimen.

Cyclic Loading and Unloading Tests: Cyclic loading and unloading tests with varying strains and recovery durations were carried out using the same apparatus. Specifically, the dumbbell-shaped specimens were stretched to a maximum strain of 700% and subsequently unloaded to the initial state at a constant strain rate of 0.14 s^−1^. For the time-dependent tests, successive loading–unloading cycles were conducted with recovery times ranging from 0 to 10 min between cycles.

Puncture Resistance Test: Hydrogel specimens (50 × 20 × ~0.7 mm thick) were placed on a sharp nail and manually pressed downward. The puncture resistance was assessed by observing the largest deformation before the point of rupture.

Drying Rate Measurement: 2 mL of the homogeneous lignin/PDMA precursor solution was added dropwise onto a steel plate. The solution was allowed to spread naturally on the thermoconductive substrate to form a thin layer. Subsequently, the steel plate was transferred to a heating stage at various temperatures. The mass variation was recorded every 10 min until a constant weight was achieved.

Peeling Test: A 90° peeling test was performed on different substrates (glass, PTFE and steel plates) using the aforementioned tensile testing system. To prevent peeling energy errors induced by tensile deformation of the hydrogel, a fiberglass cloth was used as a non-stretchable backing material. Specifically, 2 mL of the homogeneous lignin/PDMA precursor solution (118.75 mg mL^−1^) was applied onto the substrate and dried to form the first xerogel layer. Subsequently, 1 mL of the same precursor solution was added on the xerogel layer, and a fiberglass cloth was embedded into this liquid as the second layer. After solvent evaporation, these coatings along with the substrates were placed in water until fully swollen. The final swollen hydrogel coating had dimensions of 60 × 20 × ~0.3 mm and the test was conducted at a constant peeling speed of 10 mm min^−1^.

## Data Availability

The data presented in this study are available on request from the corresponding author.

## Supplementary Materials

The following supporting information can be downloaded at: https://www.mdpi.com/article/10.3390/gels12040277/s1, Table S1. Hansen Solubility Parameters of lignin and various solvents; Table S2. Guinier plot fitting parameters within *q*_min_*R*g < 0.65 and *q*_max_*R*g < 1.0; Table S3. Broad peak fitting parameters of lignin/PDMA hydrogels; Table S4. Water content of Lignin/PDMA hydrogels; Table S5. Mechanical and energy dissipative properties of lignin/PDMA hydrogels; Table S6. Peeling energy of Lignin/PDMA hydrogel coatings on different substrates; Figure S1. Solubility test of PDMA in different solvents; Figure S2. (a) 1D SAXS profiles and (b–f) broad peak simulations for different Lignin/PDMA hydrogels; Figure S3. Time-dependent cyclic tensile curves of different samples under 700% strain: (a) LG-D; (b) LG-DA-3:1; (c) LG-DA-2:1; (d) LG-DA-1:1; (e) LG-DA-1:2; Figure S4. Cyclic tensile curves of LG-DA-1:2 at various strains with a waiting time dependence; Figure S5. Solubility comparison of lignin and PDMA in water.
