# Supplementary material for: Solvent-Driven Nanostructural Tuning of Lignin/Poly(N,N-dimethylacrylamide) Hydrogels
Source: Gels. 2026 Mar 26;12(4):277. doi: 10.3390/gels12040277 (PMC13116357; doi:10.3390/gels12040277)
Supplement: Supplementary file 1 [file gels-12-00277-s001.zip › gels-4207291-supplementary.pdf]

# Solvent-Driven Nanostructural Tuning of Lignin/ poly(*N,N*-dimethylacrylamide) Hydrogels

**Xiaoqing Jiang [1], Xiangyu You [1,*], Xinhong Li [1], Ruiyun Tian [1], Xuelian Wang [2], Pedram Fatehi [3], Kang Kang [3], Xulong Zhu [4] and Huijie Zhang [1,*]**

[1] College of Bioresources Chemical and Materials Engineering, Shaanxi University of Science & Technology, Xi'an, Shaanxi, 710021, China

[2]. School of Food and Liquor Engineering, Sichuan University of Science & Engineering, Yibin, Sichuan, 644000, China

[3]. Green Processes Research Centre and Department of Chemical Engineering, Lakehead University, Thunder Bay, Ontario P7B5E1, Canada

[4]. The Key Laboratory of Biomedical Information Engineering of Ministry of Education, School of Life Science and Technology, Xi'an Jiaotong University; Xi'an 710049, P. R. China

* Corresponding author: xyyou@sust.edu.cn (X. You)
hjzhang@sust.edu.cn (H. J. Zhang)

**Table S1**. Hansen Solubility Parameters of lignin and various solvents.

| Solvent | $\delta_D$ [MPa$^{0.5}$] | $\lvert\Delta\delta_D\rvert$ [2] | $\delta_P$ [MPa$^{0.5}$] | $\lvert\Delta\delta_P\rvert$ [3] | $\delta_H$ [MPa$^{0.5}$] | $\lvert\Delta\delta_H\rvert$ [4] | *Ra* | *Ra*/*R*$_0$ |
|---|---|---|---|---|---|---|---|---|
| Acetic acid lignin (in this study) | 20.0 | | 9.1 | | 8.5 | | | |
| *N,N*-dimethylformamide | 17.4 | 2.6 | 13.7 | 4.6 | 11.3 | 2.8 | 7.49 | 0.92 |
| Acetone | 15.5 | 4.5 | 10.4 | 1.3 | 7.0 | 1.5 | 9.22 | 1.14 |
| DA-3:1 [1] | 16.9 | 3.1 | 12.9 | 3.8 | 10.2 | 1.7 | 7.42 | 0.92 |
| DA-2:1 [1] | 16.7 | 3.3 | 12.6 | 3.5 | 9.9 | 1.4 | 7.48 | 0.92 |
| DA-1:1 [1] | 16.5 | 3.5 | 12.1 | 3.0 | 9.2 | 0.7 | 7.72 | 0.95 |
| DA-1:2 [1] | 16.1 | 3.9 | 11.5 | 2.4 | 8.4 | 0.1 | 8.10 | 1.00 |
| DA-1:3 [1] | 16.0 | 4.0 | 11.2 | 2.1 | 8.1 | 0.4 | 8.34 | 1.03 |
| Dioxane | 19.0 | 1.0 | 1.8 | 7.3 | 7.4 | 1.1 | 7.65 | 0.94 |
| Toluene | 18.0 | 2.0 | 1.4 | 7.7 | 2.0 | 6.5 | 10.84 | 1.34 |
| Ethyl acetate | 15.8 | 4.2 | 5.3 | 3.8 | 7.2 | 1.3 | 9.31 | 1.15 |
| Ethanol | 15.8 | 4.2 | 8.8 | 0.3 | 19.4 | 10.9 | 13.77 | 1.70 |
| Pyridine | 19.0 | 1.0 | 8.8 | 0.3 | 5.9 | 2.6 | 3.29 | 0.41 |
| Chloroform | 17.8 | 2.2 | 3.1 | 6.0 | 5.7 | 2.8 | 7.95 | 0.98 |
| Tetrahydrofuran | 16.8 | 3.2 | 5.7 | 3.4 | 8.0 | 0.5 | 7.26 | 0.90 |
| Methanol | 15.1 | 4.9 | 12.3 | 3.2 | 22.3 | 13.8 | 17.23 | 2.13 |

[1] DA-x:y denotes the volume ratio of DMF to acetone.

[2] $\lvert\Delta\delta_D\rvert$ denotes the absolute difference in the dispersive solubility parameters between lignin and different solvents.

[3] $\lvert\Delta\delta_P\rvert$ denotes the absolute difference in the polar solubility parameters between lignin and different solvents.

[4] $\lvert\Delta\delta_H\rvert$ denotes the absolute difference in the hydrogen-bond solubility parameters between lignin and different solvents.

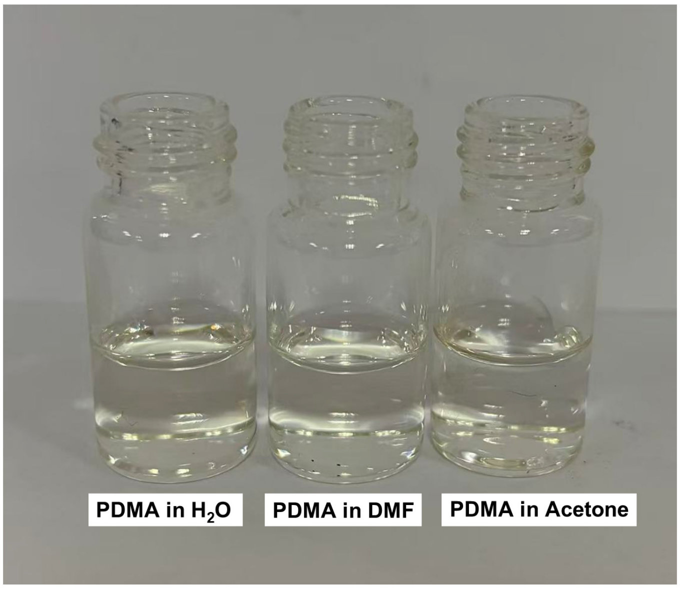


**Figure S1.** Solubility test of PDMA in different solvents.

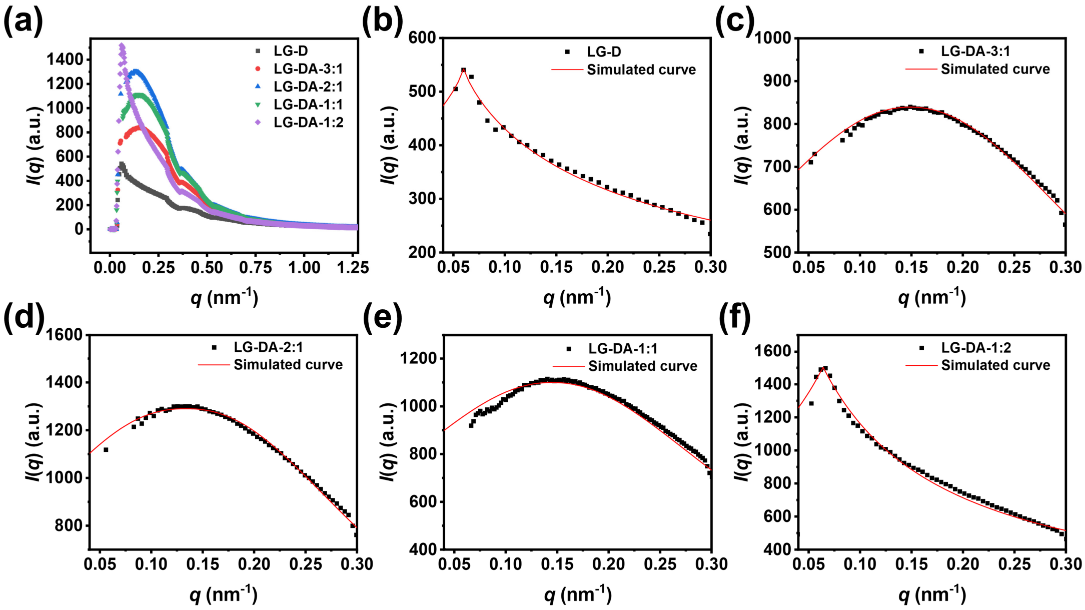


**Figure S2.** (a) 1D SAXS profiles and (b–f) broad peak simulations for different Lignin/PDMA hydrogels.

**Table S2.** Guinier plot fitting parameters within $q_{\min}Rg < 0.65$ and $q_{\max}Rg < 1.0$.

| Sample | $I(q) = a+kq$ | | $q$ range | $R^2$ | $R$g [nm] |
|---|---|---|---|---|---|
| | Slope ($k$) | Intercept ($a$) | | | |
| LG-D | -6.8 | 6.1 | 0.14-0.22 | 0.99 | 4.5 |
| LG-DA-3:1 | -4.0 | 6.8 | 0.14-0.25 | 0.97 | 3.5 |
| LG-DA-2:1 | -5.7 | 7.3 | 0.14-0.23 | 0.99 | 4.1 |
| LG-DA-1:1 | -4.8 | 7.1 | 0.14-0.25 | 0.97 | 3.8 |
| LG-DA-1:2 | -19.6 | 7.2 | 0.10-0.13 | 0.98 | 7.7 |

**Table S3.** Broad peak fitting parameters of lignin/PDMA hydrogels.

| Sample | $I(q)=C/(1+(\lvert q-q_0\rvert \xi)^m)+B$ | | | | | $d$-spacing [nm] |
|---|---|---|---|---|---|---|
| | $C$ [1] | $q_0$ | $\xi$ [2] | $m$ [3] | $B$ [1] | |
| LG-D | 540 | 0.060 | 6.5 | 1.2 | 1.0 | 104.7 |
| LG-DA-3:1 | 835 | 0.149 | 4.4 | 2.1 | 4.5 | 42.2 |
| LG-DA-2:1 | 1290 | 0.133 | 6.0 | 2.3 | 15.0 | 47.1 |
| LG-DA-1:1 | 1100 | 0.145 | 5.5 | 2.3 | 17.0 | 43.3 |
| LG-DA-1:2 | 1500 | 0.064 | 8.2 | 1.0 | 4.3 | 98.1 |

[1] $C$ and $B$ are $q$-independent constants obtained by software fitting from $I\ (q\xi \rightarrow 0)$ = C+B and $I\ (q\xi \rightarrow \infty)$ = B.

[2] $\xi$ denotes the correlation length obtained using software fitting.

[3] $m$ denotes the Porod exponent obtained using software fitting.

**Table S4.** Water content of Lignin/PDMA hydrogels.

| Sample | Water content (%) |
|---|---|
| LG-D | 66.2±0.6 |
| LG-DA-3:1 | 63.0±0.2 |
| LG-DA-2:1 | 59.1±0.6 |
| LG-DA-1:1 | 58.7±0.6 |
| LG-DA-1:2 | 56.5±0.5 |

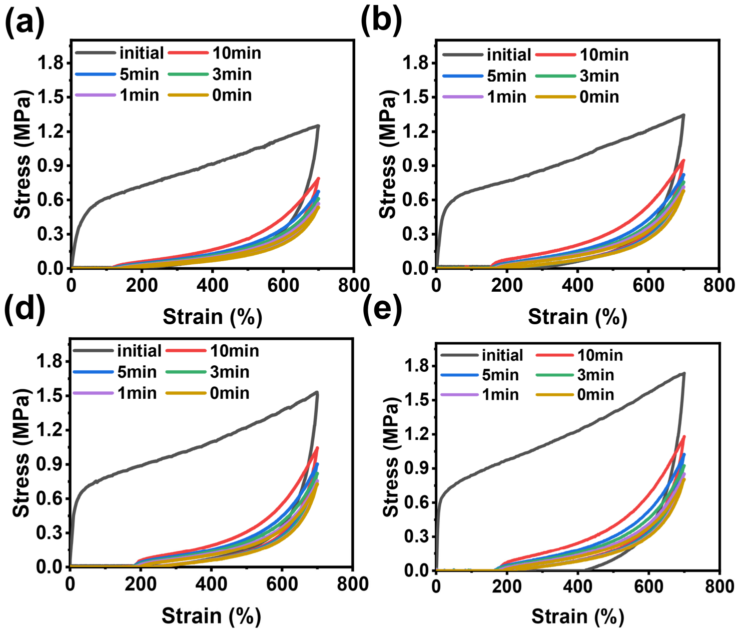


**Figure S3.** Time-dependent cyclic tensile curves of different samples under 700% strain: (a) LG-D; (b) LG-DA-3:1; (c) LG-DA-2:1; (d) LG-DA-1:1; (e) LG-DA-1:2.

**Table S5.** Mechanical and energy dissipative properties of lignin/PDMA hydrogels.

| Sample | Tensile strength [MPa] | Young's Modulus [MPa] | Fracture energy [kJ $m^{-2}$] | Hysteresis loss [MJ $m^{-3}$] |
|---|---|---|---|---|
| LG-D | 2.5±0.1 | 2.3±0.2 | 21.1±2.8 | 5.0±0.2 |
| LG-DA-3:1 | 2.7±0.1 | 2.9±0.1 | 24.9±2.1 | 5.4±0.1 |
| LG-DA-2:1 | 2.8±0.1 | 3.9±0.1 | 33.1±0.4 | 6.2±0.1 |
| LG-DA-1:1 | 2.9±0.1 | 4.2±0.1 | 36.0±2.0 | 6.8±0.1 |
| LG-DA-1:2 | 3.2±0.1 | 5.7±0.2 | 41.2±2.0 | 7.2±0.1 |

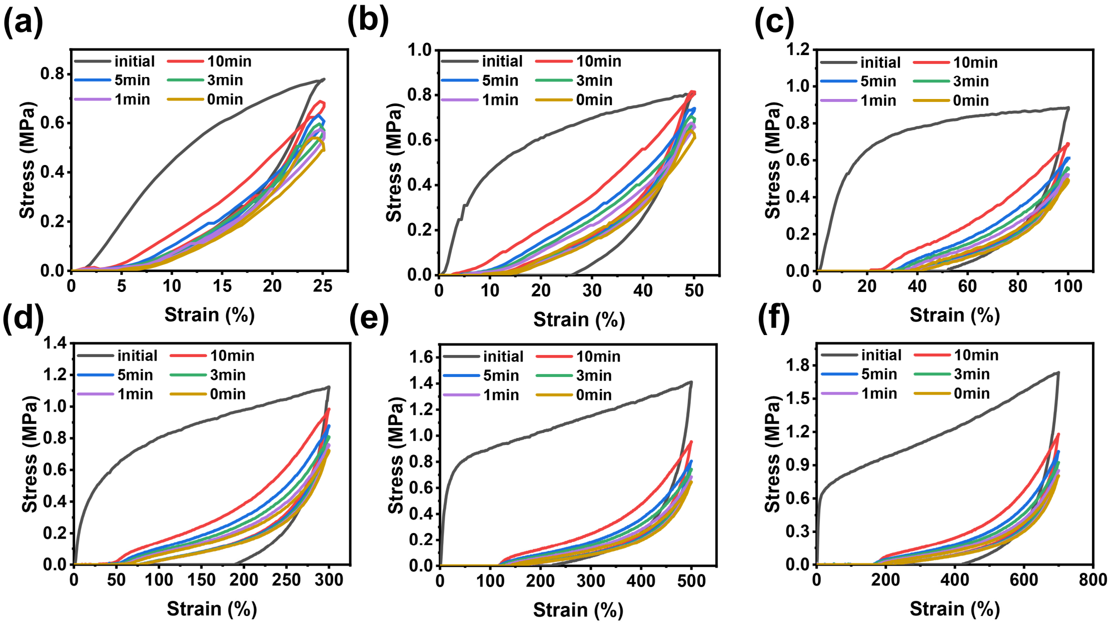


**Figure S4.** Cyclic tensile curves of LG-DA-1:2 at various strains with a waiting time dependence.

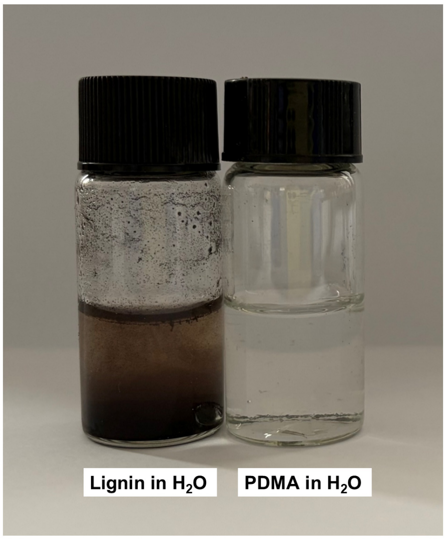


**Figure S5.** Solubility comparison of lignin and PDMA in water.

**Table S6.** Peeling energy of Lignin/PDMA hydrogel coatings on different substrates.

| Sample | Peeling energy on Glass [J m-2] | Peeling energy on PTFE [J m-2] | Peeling energy on Steel [J m-2] |
|---|---|---|---|
| LG-D | 84.2±6.3 | 119.2±12.0 | 266.0±8.3 |
| LG-DA-1:2 | 230.2±3.6 | 201.5±6.4 | 345.8±26.6 |
